# Psychiatric–Mental Health Nurse Practitioners: Addressing the Growing Mental Health Needs of the Population—A Narrative Review

**DOI:** 10.3390/healthcare14070878

**Published:** 2026-03-29

**Authors:** Yael Sela, Keren Grinberg, Rachel Nissanholtz Gannot

**Affiliations:** 1 Department of Nursing Sciences, Faculty of Social and Community Sciences, Ruppin Academic Center, Emek Hefer 4025000, Israel; kereng@ruppin.ac.il; 2 Department of Health Systems Management, Ariel University, Ariel 4076405, Israel; rachelng@ariel.ac.il

**Keywords:** psychiatric–mental health nurse practitioner, advanced practice nursing, mental health workforce, scope of practice, implementation, narrative review

## Abstract

**Highlights:**

**What are the main findings?**

- International evidence, particularly from primary care and community-based settings, suggests that PMHNP/NP-delivered care may achieve comparable outcomes on selected quality and safety indicators while improving access, continuity, and patient satisfaction.
- Successful PMHNP implementation depends not only on regulation, but also on organizational readiness, interprofessional collaboration, and clear role definition.

**What are the implications of the main findings?**

- Strategic integration of PMHNPs may strengthen mental health service capacity in Israel amid growing population needs and workforce shortages.
- Policymakers and health system leaders should align regulation, training, and organizational structures to ensure effective and sustainable utilization of the PMHNP role.

**Abstract:**

**Background:** Mental health needs are rising globally, while workforce shortages constrain access to timely care. Israel launched formal training for Psychiatric–Mental Health Nurse Practitioners (PMHNPs) in 2023 as part of broader efforts to strengthen the public mental health system. This narrative review provides a focused synthesis of international and Israeli literature on PMHNP roles, models of practice, outcomes, and implementation considerations relevant to the Israeli context. **Methods:** We conducted a narrative, non-systematic literature review of international and Israeli literature on Psychiatric–Mental Health Nurse Practitioners (PMHNPs). Searches were conducted in PubMed/MEDLINE, CINAHL, PsycINFO, and Scopus (January 2000–December 2024), alongside targeted policy and regulatory documents. Eligible sources addressed NP/PMHNP roles, scope of practice, clinical and service outcomes, implementation processes, workforce implications, or policy considerations in high-income health systems. Findings were synthesized thematically. **Results:** Across the reviewed literature, particularly in primary care and community-based settings, PMHNP/NP-delivered care was generally associated with comparable outcomes on selected quality and safety indicators, alongside improved accessibility, continuity, and high patient satisfaction. Successful implementation depended on regulatory clarity, organizational readiness, interprofessional collaboration, and the development of a clear professional identity. In Israel, the role is emerging within a cautious regulatory framework and may face early barriers related to role ambiguity, variable organizational support, and limited stakeholder awareness. **Conclusions:** PMHNP implementation may offer an important strategy for strengthening mental health service capacity in Israel. However, the extent of its contribution will depend on regulatory clarity, organizational support, implementation quality, and future empirical evaluation in the Israeli context.

## 1. Introduction

Mental health is a fundamental component of individual well-being and social functioning. Nevertheless, the provision of mental healthcare, particularly within public healthcare systems, faces substantial and persistent challenges. Global epidemiological data indicates a continuous rise in the prevalence of mental disorders, accompanied by a growing social and economic burden on individuals, families, and healthcare systems [1]. Mental health conditions are now among the leading contributors to disability worldwide, with long-term consequences for quality of life and healthcare utilization [2].

International policy reports emphasize that mental health systems in many countries are struggling to respond adequately to increasing demand due to chronic workforce shortages, fragmented service delivery, and prolonged underinvestment. These reports consistently call for structural reforms, including the expansion and optimization of advanced clinical roles, in order to improve accessibility, continuity, and quality of care [3,4].

In Israel, these global challenges are particularly pronounced. The public mental health system has long been characterized by limited resources, shortages of trained professionals, and structural disparities compared with other high-income countries [5]. Long waiting times, insufficient community-based services, and uneven geographic distribution of mental health professionals have been persistent features of the system even prior to recent crises.

The psychological consequences of the events of 7 October and the subsequent “Iron Swords” war have further intensified the demand for mental health services. The need for psychological support has increased not only in terms of acute and immediate interventions but also in relation to long-term therapeutic care for trauma-related conditions, anxiety, depression, and complex psychosocial distress [3,5]. These developments have exposed existing structural gaps and underscored the urgent need to strengthen and expand responses within the public mental health system.

As part of strategies promoted by the Israeli Ministry of Health, formal training for Psychiatric–Mental Health Nurse Practitioners (PMHNPs) was launched in 2023. This role grants nurses selected medical authorities and is intended to support the mental health system in addressing growing population needs. The introduction of a new professional role particularly in a sensitive and complex field such as mental health is a dynamic and resource-intensive process that requires time, regulatory clarity, organizational readiness, and collaboration among multiple stakeholders.

Evidence from previous initiatives in Israel suggests that the implementation of advanced nursing roles is not without difficulty. Studies examining Nurse Practitioner (NP) roles in geriatrics and supportive care have identified challenges related to role ambiguity, professional boundaries, and organizational integration [5]. These experiences highlight the importance of examining the PMHNP role not only in terms of clinical effectiveness, but also through a broader lens that considers implementation processes, system-level implications, and policy context.

Despite growing international recognition of advanced practice nursing roles, the literature specific to Psychiatric–Mental Health Nurse Practitioners remains fragmented across outcome studies, workforce analyses, regulatory documents, and implementation reports. As a result, there is limited integrative synthesis that brings together clinical effectiveness, scope-of-practice variation, organizational readiness, and policy implications in a way that is relevant for health-system planning. This gap is particularly important for Israel, where the PMHNP role has only recently been introduced and where decisions regarding implementation must be informed not only by clinical evidence, but also by international experience regarding regulation, interprofessional collaboration, and service organization. Accordingly, the present review aimed to provide a focused narrative synthesis of these interconnected domains. The review focused on literature addressing clinically deployed NP/PMHNP roles, implementation processes, service outcomes, and regulatory or policy issues in health-system contexts, rather than educational curricula alone or purely opinion-based discussions.

## 2. Materials and Methods

### 2.1. Design

This study was conducted as a narrative literature review to provide an integrative and critical overview of the role, implementation, and contribution of Nurse Practitioners (NPs), with a specific focus on Psychiatric–Mental Health Nurse Practitioners (PMHNPs), within mental health systems internationally and in Israel. A narrative approach was selected to support conceptual synthesis and contextual interpretation of clinical, organizational, and policy dimensions that may be underrepresented in strictly effectiveness-focused systematic reviews. A narrative review was considered more appropriate than a systematic or scoping review because the aim of the present study was not to estimate pooled effectiveness or map all published literature exhaustively, but rather to integrate heterogeneous evidence spanning clinical outcomes, regulatory frameworks, organizational factors, and policy considerations. This approach is consistent with published methodological guidance for narrative reviews and with quality principles reflected in the SANRA framework [6,7].

### 2.2. Literature Search Strategy

A structured literature search was conducted across PubMed/MEDLINE, CINAHL, PsycINFO, and Scopus to identify relevant peer-reviewed publications. The search covered the period from January 2000 to December 2024, reflecting the timeframe during which advanced practice nursing roles—and particularly the PMHNP role—expanded substantially in policy, practice, and research. To capture implementation and policy context, we also screened reference lists of key review articles and conducted targeted searches for regulatory and policy documents from recognized organizations (e.g., World Health Organization, OECD) and Israeli Ministry of Health circulars.

Search terms were adapted to the indexing structure and search functions of each database, combining controlled vocabulary where applicable with free-text terms. The search logic linked role-related terms (e.g., “Nurse Practitioner”, “Psychiatric–Mental Health Nurse Practitioner”, “PMHNP”) with mental health context terms (e.g., “mental health care”, “psychiatric care”, “community mental health”) and implementation or policy-related terms (e.g., “scope of practice”, “role implementation”, “advanced nursing practice”, “primary care”, and “health systems”). Reference lists of key articles and reviews were also manually screened to identify additional relevant sources. Full database-specific search strings are provided in Supplementary Table S1.

Study Selection. Eligible sources included peer-reviewed articles published in English that addressed NP or PMHNP roles in mental health care, clinical and service outcomes, implementation processes, workforce implications, or policy and regulatory considerations. Both empirical studies and review articles were included, alongside relevant policy and regulatory analyses from recognized international and Israeli organizations. Sources focused exclusively on educational curricula without practice implications, opinion pieces without empirical or policy grounding, and non-healthcare settings were excluded. Screening and source selection were performed by one reviewer, based on the predefined eligibility criteria.

Findings were synthesized thematically rather than quantitatively, emphasizing conceptual integration and relevance to implementation and health-system decision-making. Given the heterogeneity of evidence across study designs, settings, and source types, the analysis focused on identifying recurring patterns, areas of convergence, and context-specific considerations relevant to PMHNP role development and implementation. The synthesis was organized around four overarching domains: (1) the broader development and evidence base of Nurse Practitioner roles; (2) international evidence on PMHNP models of care and practice; (3) scope-of-practice and regulatory variation, interprofessional dynamics, and organizational readiness across health systems; and (4) implementation challenges and opportunities in the Israeli context. Reporting was guided by established quality principles for narrative reviews, including those reflected in the SANRA framework.

Selection Summary. The literature search and targeted document review initially identified approximately 145 records and documents. After title/abstract-level relevance screening and removal of clearly ineligible items, approximately 37 full-text sources were assessed for eligibility and relevance. Following full-text assessment, 21 sources were included in the final narrative synthesis. These comprised systematic reviews, primary empirical studies, policy and ministerial documents, and professional standards. Because this review was designed as a narrative review rather than a formal systematic review, these counts are presented to enhance transparency rather than as part of a PRISMA-based selection process. An evidence map of the included literature, organized by source category, setting, supported claims, and addressed outcomes, is provided in Supplementary Table S2.

## 3. Results

The findings are presented across four interrelated thematic domains: (1) the broader development and evidence base of NP roles; (2) international evidence regarding PMHNP models of practice; (3) scope-of-practice and regulatory variation across health systems; and (4) the Israeli context, including regulation, implementation challenges, and anticipated system implications. Throughout the review, a distinction is maintained between evidence derived from broader NP literature and evidence derived specifically from PMHNP-focused studies. This distinction is important because the broader NP evidence base is informative for role development and system planning, but it is not fully equivalent to evidence specific to psychiatric–mental health practice.

### 3.1. Nurse Practitioners: Role Development and Evidence Base

Challenges related to workforce shortages, demographic change, and the increasing burden of chronic illness are shared by healthcare systems worldwide. In response, many countries have redefined professional roles to optimize the use of available human resources and enhance service delivery [8]. One of the most prominent roles developed in this context is that of the Nurse Practitioner (NP).

The International Council of Nurses defines the Nurse Practitioner as a registered nurse who has acquired advanced clinical expertise, complex decision-making skills, and expanded professional competencies, with a master’s degree recommended for entry-level preparation [9]. Admission requirements for NP training typically include advanced academic education, formal clinical specialization, and extensive professional experience in assessment and management of defined health conditions.

The NP role originated in the United States in 1965, with the establishment of the first academic training program at the University of Colorado. Since then, the role has expanded globally and is now formally recognized and regulated in countries such as the United States, Canada, Australia, and several European nations. Despite this shared foundation, there is considerable international variation in scope of practice, level of autonomy, prescribing authority, and regulatory frameworks [9,10].

A growing body of evidence from completed systematic reviews and primary studies supports the quality and safety of NP-led care across diverse healthcare settings. Systematic reviews have shown that NP-delivered care is generally associated with comparable outcomes on selected quality indicators, with high patient satisfaction and, in some settings, reduced healthcare utilization and costs. In primary care, these effects appear particularly relevant for patients with chronic and complex conditions. However, much of this literature concerns broader NP roles rather than PMHNP-specific roles, and this distinction should be considered when interpreting implications for mental health systems [11]. In primary care settings, NP-led models have been shown to be particularly effective for patients with complex and chronic conditions. A systematic review focusing on individuals with multimorbidity found that NP-led care was associated with comparable or reduced healthcare costs, similar or improved quality of care, and reduced utilization of emergency and inpatient services [12]. These findings highlight the potential contribution of NPs to healthcare system efficiency, particularly in publicly funded systems facing increasing demand and limited physician availability.

Beyond clinical outcomes, studies have consistently reported high levels of patient satisfaction with NP-provided care, as well as positive effects on care continuity and access [13,14]. Evidence also suggests that NP integration does not result in increased referrals or unnecessary utilization of additional services, further supporting the role’s contribution to sustainable healthcare delivery [14,15,16].

Psychiatric–Mental Health Nurse Practitioners: International Evidence and Models of Practice.

Mental health systems worldwide have increasingly turned to Psychiatric–Mental Health Nurse Practitioners (PMHNPs) as a response to growing service demand, workforce shortages, and the need for accessible, community-based care. The PMHNP role is typically characterized by advanced clinical training in psychiatric assessment, diagnosis, psychotherapy, psychopharmacology, and care coordination, enabling practitioners to function with a high degree of clinical responsibility [3,17].

### 3.2. PMHNPs in Primary Care and Community Settings

One of the most extensively studied areas of PMHNP practice is primary care and community mental health services, where early identification and intervention are critical. In these settings, PMHNPs often serve as first-line providers for individuals presenting with common mental health conditions such as depression, anxiety disorders, and substance use disorders. Their integration into primary care teams has been associated with improved access to mental health services, reduced waiting times, and enhanced continuity of care [11,18,19].

Empirical studies and reviews consistently demonstrate that PMHNP-led care in community settings achieves clinical outcomes comparable to those of psychiatrist-led models. A systematic review examining NP-delivered care for individuals with depression, anxiety, and substance use disorders found no significant differences in symptom reduction, safety outcomes, or rates of hospitalization compared with physician-led care, while highlighting improved accessibility and patient satisfaction. Similar findings were reported in later analyses focusing specifically on PMHNP roles within integrated care models [18].

In addition to direct clinical outcomes, PMHNPs in community settings frequently play a central role in care coordination and case management, particularly for individuals with complex psychosocial needs. This includes collaboration with primary care physicians, social workers, psychologists, and community services, contributing to a more holistic and patient-centered approach. Such models are particularly relevant in public health systems, where fragmentation of care has been identified as a major barrier to effective mental health service delivery [19]. The strongest support for comparability comes from systematic reviews and higher-level evidence conducted mainly in primary care and community-based mental health settings. Across these studies, the best-supported outcomes include patient satisfaction, access-related measures, continuity of care, and selected clinical outcomes such as symptom improvement and hospitalization-related indicators. However, the evidence base is less uniform across all mental health service contexts, and some findings derive from broader NP literature rather than PMHNP-specific studies alone. In addition, for Israel, the relevance of these findings remains partly indirect, as local empirical outcome data on PMHNP implementation are not yet available.

### 3.3. PMHNPs in Inpatient and Specialized Services

Beyond community-based care, PMHNPs are increasingly integrated into inpatient psychiatric units and specialized mental health services. In these settings, their roles often include comprehensive psychiatric assessments, medication management, participation in multidisciplinary treatment planning, and follow-up care planning. Studies examining inpatient PMHNP practice suggest that their involvement contributes to improved continuity between inpatient and community services, as well as more efficient use of psychiatric resources.

Workforce data from several high-income countries indicate a substantial expansion of the PMHNP workforce over the past decade, coinciding with a relative decline in the number of practicing psychiatrists in public systems [20]. This trend has intensified interest in optimizing the utilization of PMHNPs across care settings, particularly in regions experiencing acute shortages of psychiatric physicians.

### 3.4. Scope of Practice and Regulatory Variation

Despite shared core competencies, the scope of PMHNP practice varies considerably across countries and jurisdictions. In some systems, PMHNPs are granted full practice authority, including independent prescribing and autonomous clinical decision-making. In others, practice authority remains conditional upon physician supervision or collaborative agreements. Comparative analyses suggest that broader scopes of practice are associated with improved service accessibility and workforce stability, without compromising quality or safety [21].

Nevertheless, regulatory expansion alone does not guarantee effective role implementation. Organizational culture, interprofessional relationships, and clarity of role definition have been identified as critical determinants of successful PMHNP integration. These factors underscore the importance of examining PMHNP practice not only through a clinical lens, but also in relation to system-level and organizational contexts.

Psychiatric–Mental Health Nurse Practitioners in Israel: Context, Regulation, and Implementation.

The introduction of the PMHNP role in Israel represents a significant development within the national mental health system. Historically, advanced nursing roles in Israel have been implemented gradually and unevenly, often accompanied by professional and organizational challenges. Understanding this context is essential for evaluating the potential contribution of PMHNPs and anticipating barriers to effective integration.

### 3.5. Development of Advanced Nursing Roles in Israel

Advanced nursing practice in Israel has evolved primarily through targeted initiatives addressing specific system needs, particularly in geriatrics, palliative care, and other selected areas of workforce shortage. While these initiatives have demonstrated the potential of advanced practice roles to enhance service delivery, Israeli experience has also highlighted implementation challenges related to role definition, practice authority, stakeholder awareness, and organizational integration [22].

These experiences provide an important backdrop for the implementation of the PMHNP role. Unlike some countries where NP roles were introduced alongside broad regulatory reforms, Israel’s approach has often involved incremental delegation of authority, with significant discretion retained by supervising physicians and healthcare organizations.

### 3.6. Regulatory Framework for PMHNPs in Israel

According to the Ministry of Health Director-General Circular [23] (No. 4/2023), PMHNPs are formally recognized as part of multidisciplinary mental healthcare teams and are authorized to perform a defined set of clinical activities. These include psychiatric assessment, referral for diagnostic testing, management of acute and chronic psychiatric conditions, and renewal of pharmacological treatment in accordance with clinical guidelines.

Eligibility criteria for PMHNP training include completion of post-basic psychiatric nursing certification and a minimum of two years of professional experience in mental health settings. The training program comprises approximately 726 academic and clinical hours and culminates in a comprehensive examination. PMHNPs operate under dual supervision: professional supervision by a specialist physician and administrative supervision by nursing management. Importantly, the exercise of medical authority is subject to physician approval, and implementation of granted powers is not mandatory across settings. This regulatory structure reflects a cautious approach to role expansion, aimed at balancing innovation with professional oversight.

### 3.7. Early Implementation Challenges

While empirical data on PMHNP practice in Israel remain limited due to the recency of the role, evidence from other NP specialties suggests that similar challenges are likely to emerge. These include uncertainty regarding role boundaries, variability in organizational support, and limited awareness of NP competencies among physicians and managers [22,23].

In the context of mental health, such challenges may be further compounded by high workload, staff shortages, and the emotional demands of psychiatric care [3]. Without clear implementation strategies and organizational commitment, there is a risk that PMHNPs will be underutilized, with their roles confined to functions already performed by post-basic psychiatric nurses rather than fully leveraging their advanced training.

**Figure 1:**
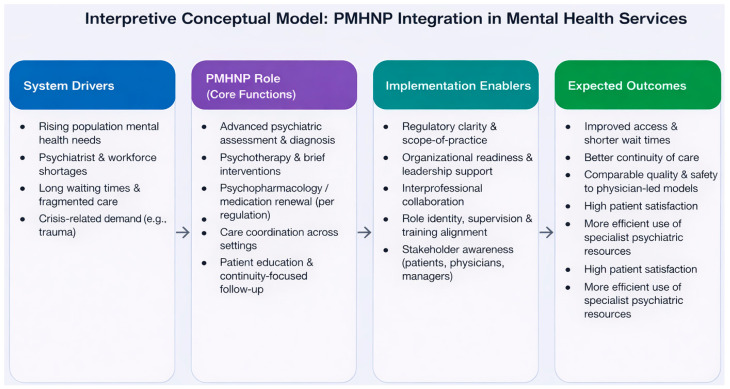
Conceptual synthesis model derived from the reviewed literature, linking system drivers, core PMHNP role functions, implementation enablers, and anticipated service and system outcomes in the Israeli mental health context. The figure is intended as a synthesis tool to organize the reviewed evidence, not as an empirically validated framework.

As the PMHNP role in Israel was introduced only recently, empirical outcome data are not yet available, and evaluation of its impact will require longitudinal and mixed-methods research. To integrate the findings of this review, Figure 1 presents an interpretive conceptual synthesis model derived from three interrelated bodies of evidence: (1) international literature on NP/PMHNP roles, outcomes, and models of care; (2) implementation-oriented literature addressing role clarity, interprofessional collaboration, and organizational readiness; and (3) Israeli policy and regulatory sources relevant to the early development of the PMHNP role. The model organizes the review findings into four linked domains: system drivers (e.g., rising mental health needs and workforce shortages), core PMHNP role functions (e.g., assessment, medication management, follow-up, and care coordination), implementation enablers (e.g., regulation, leadership support, role clarity, and collaborative practice), and expected service and system outcomes (e.g., improved access, continuity, patient satisfaction, and more efficient use of psychiatric resources). In the Israeli context, the model is intended as a policy- and implementation-oriented framework rather than an empirical outcome model.

## 4. Discussion

It is important to note that some of the evidence discussed in this review derives from the broader NP literature rather than from PMHNP-specific studies. Accordingly, implications for PMHNP implementation should be interpreted with caution, especially when extrapolating from general advanced practice nursing evidence to mental health systems.

The reviewed literature, particularly in primary care and community-based settings, suggests that PMHNPs may achieve comparable outcomes on selected quality and safety indicators, while also offering potential benefits related to accessibility, continuity, and system efficiency [13,14,20]. Recent evidence further strengthens this interpretation. A 2025 multiple-case study in general practice found that the integration of psychiatric mental health nurse practitioners was associated with improved perceived accessibility and quality of mental healthcare, greater professional satisfaction, and reduced workload for general practitioners. Importantly, these benefits appeared to depend not only on the presence of the role itself, but also on enabling conditions such as professional autonomy, trust, role clarity, and organizational support. This is consistent with broader recent evidence from advanced practice nursing, which suggests that these roles can improve access to care, reduce unnecessary service utilization, and contribute to system efficiency, particularly when embedded within supportive practice environments. Together, these findings reinforce the relevance of PMHNP implementation as a potentially meaningful strategy for strengthening mental health service delivery, while also highlighting the importance of organizational and regulatory conditions in shaping its impact [24,25].

### 4.1. Professional Identity and Role Implementation

From a theoretical perspective, the challenges associated with PMHNP integration can be understood through the lens of Role Theory, which emphasizes the impact of role expectations, role ambiguity, and role conflict on professional practice. When expectations regarding authority, responsibility, and autonomy are unclear or contested, role strain may occur, potentially limiting the effective utilization of professional competencies. Evidence from both international and Israeli contexts suggests that successful PMHNP implementation depends on the establishment of a clear professional identity within multidisciplinary teams. This includes explicit role definitions, alignment between training and practice, and recognition of PMHNP competencies by physicians and organizational leaders [23].

### 4.2. Interprofessional Dynamics and Organizational Readiness

Interprofessional collaboration is a central component of mental healthcare delivery. While PMHNPs are trained to function within multidisciplinary teams, their integration may challenge traditional professional hierarchies. Studies examining NP implementation consistently identify physician resistance and organizational inertia as key barriers, particularly in systems where medical authority has historically been centralized [25]. Organizational readiness, including leadership support, clear protocols, and opportunities for collaborative practice has been shown to facilitate successful role integration. This point is also consistent with recent implementation literature beyond the PMHNP field. A 2025 systematic review on organizational readiness for change in healthcare emphasized that successful implementation depends not only on formal authorization, but also on whether organizational members feel prepared, supported, and aligned around the change process. In the context of PMHNP integration, this suggests that role uptake is likely to depend on leadership support, local workflow adaptation, interprofessional legitimacy, and the extent to which the role is visibly embedded in routine service delivery [26]. In the absence of such conditions, PMHNPs may experience limited autonomy and reduced job satisfaction, potentially undermining workforce retention and system benefits [11,23].

PMHNP roles differ substantially in educational preparation, legal authority, reimbursement structures, and models of team-based care. Consequently, findings from jurisdictions with broad autonomous practice cannot be assumed to transfer directly to systems in which scope of practice remains more restricted. In the Israeli case, this caution is particularly important because the role is still emerging and empirical local outcome data are not yet available. Accordingly, the present review should be understood as offering an interpretive and policy-relevant synthesis rather than definitive evidence regarding the impact of PMHNP implementation in Israel.

### 4.3. Cross-Country Variation, Equity, and Access Considerations

These implementation issues should also be interpreted in light of substantial cross-country variation in PMHNP regulation, service models, and health-system organization. International literature suggests that PMHNP roles are most effective when embedded within clearly structured models of care, such as stepped-care and collaborative-care frameworks, which support early identification of mental health conditions, continuity across levels of care, and stronger integration between physical and mental health services. Within such models, PMHNPs often assume responsibility for longitudinal follow-up, medication management, and coordination between primary care and specialist services, thereby helping to reduce fragmentation of care.

Equity considerations extend beyond geographic access alone. PMHNPs may be particularly valuable for vulnerable populations who face delayed or fragmented care, including people living in disadvantaged communities and individuals with chronic or complex mental health needs. In such contexts, PMHNPs may reduce barriers to timely assessment, strengthen continuity, and support more person-centered follow-up. In Israel, future implementation should therefore be examined not only in terms of overall capacity, but also in relation to equitable access for underserved populations and peripheral regions.

Equity and access considerations are especially relevant in this context. Evidence from publicly funded health systems suggests that PMHNPs may be particularly valuable in underserved or peripheral regions, where access to psychiatrists is limited and continuity of care is difficult to maintain. Comparative analyses further suggest that systems granting broader professional autonomy to PMHNPs may achieve more efficient use of psychiatric resources, with psychiatrists focusing on diagnostically complex or treatment-resistant cases while PMHNPs manage a substantial proportion of routine and moderate-severity conditions. At the same time, the transferability of these models to Israel should be interpreted cautiously, given the country’s more restrictive regulatory framework and the current absence of local outcome data. Recent policy evidence also suggests that regulatory context may shape not only workforce utilization but also population-level outcomes. For example, a 2024 study examining Advanced Practice Registered Nurse full practice authority reported suggestive evidence of improved access to care and a reduction in poor mental health days. Although these findings derive from the U.S. policy context and cannot be transferred directly to Israel, they reinforce the argument that scope-of-practice restrictions may influence the extent to which advanced nursing roles can contribute to mental health system responsiveness [27].

### 4.4. Implications for Service Organization

From a service organization perspective, the integration of PMHNPs may contribute to reconfiguration of mental health service delivery. Rather than functioning solely as substitutes for psychiatrists, PMHNPs often introduce distinct professional perspectives grounded in nursing frameworks that emphasize holistic assessment, patient education, and psychosocial support. These contributions may be particularly relevant in community mental health settings, where social determinants of health play a central role in patient outcomes [3]. The literature also suggests that PMHNPs may enhance system responsiveness during periods of crisis. During times of increased demand, such as public health emergencies or armed conflict, flexible deployment of PMHNPs across settings may help mitigate service disruptions. Their broad clinical training enables adaptation to diverse roles, including triage, short-term interventions, and coordination of follow-up care.

### 4.5. Policy Implications

International policy documents underscore the urgency of addressing mental health workforce shortages through innovative role expansion. Both the World Health Organization and the OECD explicitly recommend optimizing the use of advanced nursing roles as part of comprehensive mental health system reform [3,4]. These recommendations are highly relevant to Israel, where rising demand, limited psychiatric workforce capacity, and recent national crises have intensified pressure on public mental health services. Strategic implementation of the PMHNP role in Israel will require more than regulatory authorization. Policy efforts should focus on aligning training, regulation, and organizational practice; promoting awareness of PMHNP competencies; and establishing evaluation mechanisms to monitor outcomes and guide ongoing development. More broadly, recent international evidence suggests that the contribution of advanced practice nursing should be evaluated not only in terms of workforce substitution, but also in relation to service redesign, continuity of care, cost containment, and equitable access for underserved populations. For Israel, this means that PMHNP implementation should be accompanied by explicit policy planning regarding organizational placement, interprofessional collaboration, performance indicators, and long-term workforce development.

### 4.6. Future Directions in the Israeli Context

Future research in Israel should move beyond descriptive policy discussion and examine the implementation of the PMHNP role through empirical, longitudinal, and mixed-methods designs. Priority outcomes should include clinical indicators, patient satisfaction, continuity of care following hospitalization, waiting times for mental health services, workforce stability, role clarity, interprofessional collaboration, and service utilization. In addition, qualitative and implementation-focused studies should examine how PMHNPs, psychiatrists, managers, and service users experience the role in practice, and which organizational and regulatory conditions support or constrain effective integration. Such research would help determine not only whether PMHNPs improve access and quality, but also under what circumstances they are most effective in the Israeli mental health system.

## 5. Limitations

This review has several limitations. First, as a narrative review, it was not designed as a formal systematic review and therefore remains subject to potential selection bias despite the use of structured database searches, predefined eligibility criteria, and supplementary policy and reference-list screening. Second, the included literature spans heterogeneous jurisdictions, regulatory frameworks, service models, and outcome measures, which limits direct comparability across studies and cautions against overly broad generalization. Third, a substantial portion of the evidence base derives from international contexts—particularly primary care and community-based settings in high-income countries—and its applicability to Israel remains partly indirect. This is especially important given that the PMHNP role is only beginning to emerge in Israel and local outcome data are not yet available. Finally, the review integrates multiple source types, including empirical studies, reviews, policy documents, and professional standards, which is appropriate for the review’s policy- and implementation-oriented purpose, but this also means that the strength of evidence is not uniform across all claims and domains discussed.

## 6. Conclusions

The reviewed literature suggests that PMHNPs may contribute to improved access, continuity, and selected quality-related outcomes when implemented within supportive regulatory and organizational environments. However, much of the available evidence derives either from broader NP literature or from international PMHNP experience, and Israeli outcome data are not yet available. Accordingly, in the Israeli context, the PMHNP role should currently be understood as a promising strategic opportunity rather than a demonstrated solution. Its ultimate contribution will depend on implementation quality, organizational support, interprofessional collaboration, and future empirical evaluation. This review contributes to the literature by integrating clinical, organizational, and policy perspectives on PMHNPs and by situating their potential role within the unique challenges currently facing the Israeli mental health system. In a context of growing unmet need and workforce constraints, timely and strategic implementation of the PMHNP role—supported by clear regulation, organizational alignment, and interprofessional collaboration—may offer an important strategic opportunity to strengthen public mental health services, although further empirical evaluation in the Israeli context is still needed.

## Data Availability

No new data were created or analyzed in this study. Data sharing is not applicable to this article.

## Supplementary Materials

The following supporting information can be downloaded at: https://www.mdpi.com/article/10.3390/healthcare14070878/s1, Table S1: Overview of the search strategy used in the narrative review; Table S2: Evidence map of the literature included in the narrative review.

## Abbreviations

The following abbreviations are used in this manuscript:

NP	Nurse Practitioner
PMHNP	Psychiatric–Mental Health Nurse Practitioner
OECD	Organisation for Economic Cooperation and Development
WHO	World Health Organization
