# Supplementary material for: Psychiatric–Mental Health Nurse Practitioners: Addressing the Growing Mental Health Needs of the Population—A Narrative Review
Source: Healthcare (Basel). 2026 Mar 29;14(7):878. doi: 10.3390/healthcare14070878 (PMC13072848; doi:10.3390/healthcare14070878)
Supplement: Supplementary file 1 [file healthcare-14-00878-s001.zip › Table S1.pdf]

Supplementary Table S1. Overview of the search strategy used in the narrative review

Database / source	Time frame	Main concepts searched	Notes
PubMed/MEDLINE	January 2000 – December 2024	Nurse Practitioner; Psychiatric–Mental Health Nurse Practitioner; PMHNP; mental health care; psychiatric care; community mental health; scope of practice; role implementation; outcomes; regulation	Search adapted to database indexing and free-text terms
CINAHL	January 2000 – December 2024	Nurse Practitioner; PMHNP; advanced nursing practice; mental health care; psychiatric care; scope of practice; workforce; continuity; access	Search adapted to nursing and allied health subject headings
PsycINFO	January 2000 – December 2024	Nurse Practitioner; PMHNP; mental health care; psychiatric care; role implementation; patient satisfaction; continuity of care	Search focused on mental health and service-delivery terminology
Scopus	January 2000 – December 2024	Nurse Practitioner; PMHNP; advanced nursing practice; mental health care; regulation; workforce; access; continuity; outcomes	Broad interdisciplinary search
Policy and regulatory documents	January 2000 – December 2024	PMHNP; Nurse Practitioner; mental health workforce; scope of practice; regulation; policy; Israel Ministry of Health; WHO; OECD	Used to identify policy, regulatory, and ministerial sources
Reference list screening	Not restricted	Reference lists of key reviews, empirical studies, and policy documents	Used to identify additional relevant sources

Note: Search terms were adapted to the indexing structure and search functions of each database. As this was a narrative, non-systematic review, the search strategy was designed to identify conceptually and policy-relevant literature rather than to support exhaustive PRISMA-based retrieval.
