# Supplementary material for: Psychiatric–Mental Health Nurse Practitioners: Addressing the Growing Mental Health Needs of the Population—A Narrative Review
Source: Healthcare (Basel). 2026 Mar 29;14(7):878. doi: 10.3390/healthcare14070878 (PMC13072848; doi:10.3390/healthcare14070878)
Supplement: Supplementary file 1 [file healthcare-14-00878-s001.zip › Table S2.pdf]

Supplementary Table S2. Evidence map of the literature included in the narrative review

Source category	Key references	Setting/country	Main claim supported	Outcomes addressed
Global epidemiological background	Xu et al., 2025 [1]; GBD 2019 Mental Disorders Collaborators, 2022 [2]	Global	Mental health disorders constitute a major and growing burden, reinforcing the need to expand service capacity	Prevalence, disability burden, social/economic impact
International policy and workforce drivers	WHO, 2022 [3]; OECD, 2023 [4]; WHO, 2016 [8]	Global / international health systems	Mental health systems face workforce shortages and service fragmentation; advanced nursing role expansion is part of the recommended system response	Workforce shortages, access pressures, system reform, service capacity
Israeli mental health system context	Rosenthal et al., 2023 [5]	Israel	Israel faces substantial structural and economic challenges in mental health care, supporting the relevance of role expansion	Service gaps, international comparison, policy need, system strain
Professional standards and role definition	International Council of Nurses, 2018 [9]; American Psychiatric Nurses Association, 2022 [17]	International; United States	Defines NP/PMHNP competencies, advanced practice expectations, and role scope in mental health care	Role definition, competencies, scope of practice, clinical responsibilities
NP role development / professional evolution	American Association of Nurse Practitioners, historical timeline [8]	United States / international relevance	The NP role has a long developmental trajectory and formal professionalization history	Role development, regulation, professional expansion
Review-level evidence on NP outcomes in general care	McMenamin et al., 2023 [12]; Laurant et al., 2018 [14]	Primarily high-income countries / primary care	NP-led care can achieve comparable quality and safety on selected indicators and may improve efficiency in chronic and primary care settings	Quality of care, safety, patient satisfaction, service utilization, cost/resource use

Source category	Key references	Setting/country	Main claim supported	Outcomes addressed
PMHNP / mental health review-level evidence	Turi et al., 2023 [18]	Primary care mental health settings	NP/PMHNP care for common mental health conditions is associated with comparable selected outcomes and improved access in primary care settings	Symptom outcomes, access, continuity, patient satisfaction
Primary empirical evidence on NP care delivery	DesRoches et al., 2017 [13]; Hing et al., 2011 [16]	United States / vulnerable populations / community health centers	NP-delivered care can maintain quality in community-facing and vulnerable-population settings	Quality indicators, access, continuity, community-based service delivery
PMHNP workforce and service models	Delaney et al., 2019 [19]; Oh et al., 2022 [20]	United States	PMHNPs are increasingly used to address psychiatrist shortages and expand service capacity across care settings	Workforce growth, psychiatrist substitution/complementarity, access, service capacity
Policy / integrated care implications for PMHNP use	Delaney et al., 2018 [26]	United States / integrated care	Effective PMHNP integration depends on policy alignment, collaborative care structures, and implementation conditions	Access, quality, integrated care, policy implications
Israeli advanced nursing implementation experience	Christianson-Silva et al., 2021 [15]; Haron et al., 2019 [24]	Israel	Previous NP implementation in Israel shows potential contribution to service delivery, but also highlights barriers such as role ambiguity, limited autonomy, and organizational readiness	Implementation barriers, professional boundaries, organizational integration, role clarity
Cross-cutting synthesis relevant to the Israeli PMHNP context	Sources across categories [3–5,8,12–14,17–23]	International + Israel	The strongest support concerns access, continuity, patient satisfaction, and selected quality/safety outcomes; transferability to Israel remains partly indirect because local PMHNP outcome data are still emerging	Access, continuity, satisfaction, selected quality/safety indicators, implementation feasibility
